# Complicated Neurotuberculosis with sinus venous thrombosis: A case-report

**DOI:** 10.1016/j.idcr.2022.e01374

**Published:** 2022-01-03

**Authors:** Yousra Ali, Yahia Imam, Hasan S. Ahmedullah, Naveed Akhtar, Saadat Kamran, Muna Al Maslmani, A. Latif Al Khal, Ahmed Own, Dirk Deleu

**Affiliations:** a Department of Medicine, Hamad Medical Corporation Doha, Qatar; b Neuroscience Institute, Hamad Medical Corporation Doha, Qatar; c Weil Cornell Medicine, Doha, Qatar; d Center for Communicable Disease, Doha, Qatar

**Keywords:** Neurotuberculosis, Tuberculous meningitis, Hydrocephalus, Infarcts, TB granuloma, Cranial nerve palsies, Cerebral venous thrombosis, Culture negative

## Abstract

**Introduction:**

Neurotuberculosis comprises around 6% of systemic tuberculosis. It targets a younger population, and it often leads to severe neurological complications or death.

**Case report:**

We report a young gentleman with a clinically defined tuberculous meningitis (TBM) and multiple neurological complication associated with TBM occurring simultaneously. This includes hydrocephalus requiring a ventriculoperitoneal shunt, vasculitic infarcts, cranial nerve palsies, TB granuloma and cerebral venous thrombosis. The cerebrospinal fluid polymerase chain reaction for tuberculosis as well as cultures remained negative repeatedly. The patient was treated with anti-tuberculous medication in addition to steroids based on validated scoring systems suggestive of TBM and made a good recovery.

**Conclusion:**

This report highlights the different complication seen with TBM and the importance of using clinical criteria to guide management plan particularly when cultures are negative.

## Introduction

Tuberculosis (TB) remains a major global health problem afflicting both the underdeveloped and the developed world alike [1]. Neuro-tuberculosis is an uncommon form of extra pulmonary TB, occurring in 1–10% of all cases of TB [2]. Tuberculous meningitis (TBM) is the commonest and severest form of neuro-tuberculosis [3].

Diagnosis of TBM is challenging due to its variable presentation and low sensitivity of the diagnostic tests. It carries high morbidity and mortality if left untreated [3].

We report a case of young gentleman with all the classical complication of TBM where microbiological tests failed to demonstrate tuberculosis and was diagnosed and treated based on clinical suspension aided by validated scoring systems.

## Case report

**Fig. 1:**
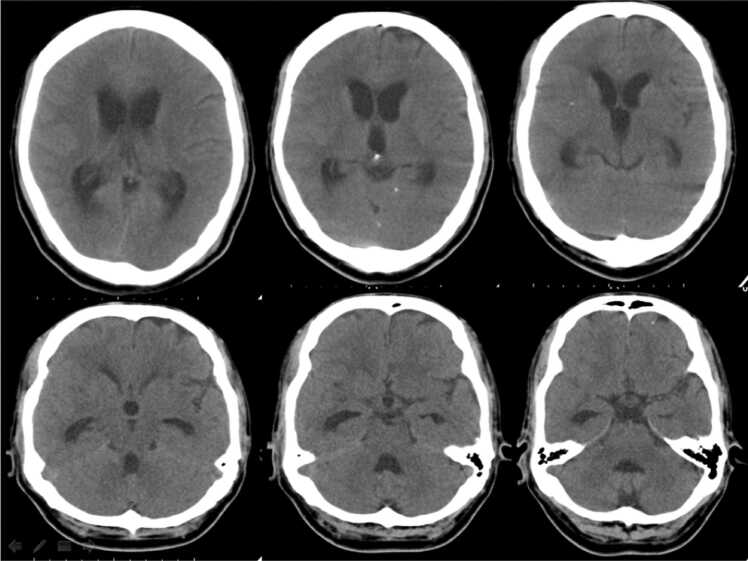
Non -contrast CT of the brain showing hydrocephalus.

**Fig. 2:**
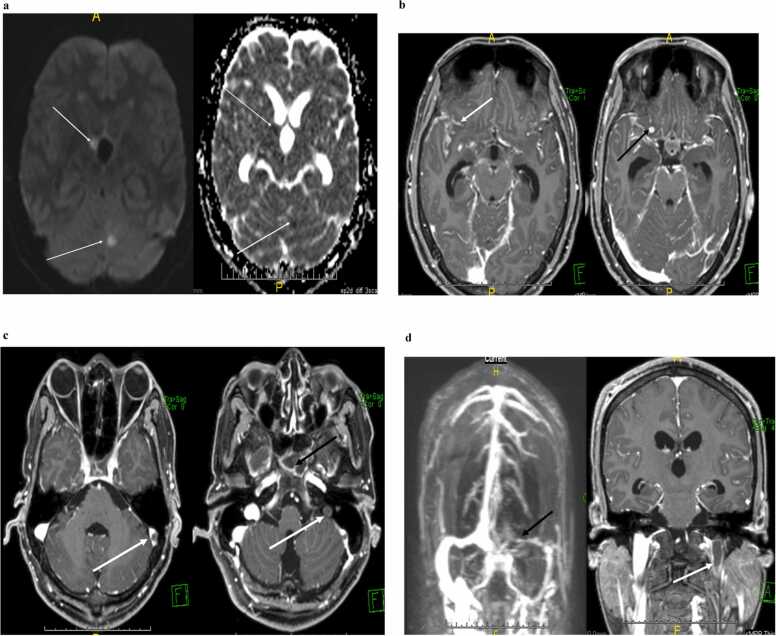
**a:** MRI of the brain showing a paired Diffusion weighted (DWI) sequence with an apparent coefficient map (ADC map) sequence indicating the vasculitic infarcts (white arrows). Note that they appear hyperintense on DWI and hypointense on ADC map indicating acuteness. **b:** Axial contrast enhanced T1 weighted MRI of the brain showing ring enhancing right frontal tuberculoma (black arrow). Note the accumulation of inflammatory exudates around the right Sullivan fissure in a gyral pattern (white arrow). **c:** Axial T1 contrast-enhanced MRI of the brain showing thrombosis of the right sigmoid sinus extending down to the jugular vein (white arrow). Note the intense basal enhancement (black arrow). **d:** Contrast enhanced MRV (left) showing filling defect in the left transverse and sigmoid sinuses as extending well into the left jugular vein (black arrow). Coronal contrast enhanced MRI of the brain: showing the extension of the thrombus well into the left jugular vein (black arrow).

A 34-year-old Nepalese male, presented with confusion and abnormal behavior of 8-days duration. No witnessed seizures were reported. Examination showed a thin man with normal vital signs. He was drowsy, needed to be prompted continuously to respond, and was echolalic. There was prominent neck rigidity and bilateral 3rd nerve palsies in addition to peripheric facial palsy on the right. Gag and palatal movement were intact. The patient could move all 4 limbs equally, had bilateral extensor planter responses and an unsteady gait. Other systemic examination was unremarkable. Complete blood count, coagulation profile and serum chemistry were all within normal limits. HIV test was negative. A non-contrast computed tomography (CT) of the brain (Fig. 1) revealed hydrocephalus necessitating the insertion of an emergent external ventricular device (EVD) which was changed later to a ventriculoperitoneal shunt. CSF examination showed predominately lymphocytic inflammatory picture with around 330 cells, a moderately elevated protein of 0.75 g/l and a severely depressed glucose of 0.5 mmol/l. CSF Gram staining and cultures were negative. Polymerase chain reaction (PCR) for TB as well as Acid fast bacilli (AFB) staining, and TB culture was negative on 3 different occasions. CSF viral serology was also negative. Magnetic resonance imaging (MRI) with contrast including a MR venogram (MRV) (Fig. 2a–d) showed extensive basal enhancement, a solitary ring enhancing lesion (likely tuberculoma), left transverse and sigmoid sinus thrombosis extending to the jugular vein and two discrete vasculitic infarcts in addition to the presence of hydrocephalus with an EVD in place. Based on a score of – 5 on the Thwaites Diagnostic Score (TDS) [4] and a score of 14 on the Lancet Consensus score (LCS) [5] the patient was diagnosed with probable TBM and started on antituberculosis treatment (ATT) using the standard 4 drug regimen of rifampicin, isoniazid, ethambutol and pyrazinamide in weight adjusted doses in addition to dexamethasone. Further work up including a mantoux skin test, chest x-ray, an autoimmune screening, and a thrombophilia screening had a negative yield. The patient was not commenced on anticoagulants as the risk of bleeding with EVD in place was deemed high.

Patient showed good recovery with restoration of normal cognitive function, but residual right sided oculomotor and facial palsies persisted. CSF showed reduction of cells and protein and normalization of CSF glucose after 20 days of treatment. The patient was sent to rehabilitation on ATT where he continued to improve and was discharged home after 2 months of rehabilitation with the above-mentioned deficits. The patient thereafter traveled back to his country and was lost to follow up.

## Discussion

Out of all systemic TB cases, 2–5% suffer the complications of CNS TB. This number increases to 10% in patients with HIV co-infection. The spectrum of CNS TB varies widely, commonly manifesting in the brain as tuberculous meningitis (the severest and commonest form of neuro-tuberculosis), tuberculous abscess, tuberculoma [6] or rarely as hypertrophic pachymeningitis. And in the spinal cord it manifests as tuberculous myelitis, spinal tuberculoma, Pott’s spine with epidural abscess and arachnoiditis [7]. Other forms of the disease are further classified in Table 1.

**Table 1:** [4] Classification of CNS tuberculosis.

**Intracranial**
Tuberculous meningitis (TBM)
TBM with miliary tuberculosis
Tuberculous encephalopathy
Tuberculous vasculopathy
CNS tuberculoma (single or multiple)
Tuberculous abscess
**Spinal**
Pott’s spine and Pott’s paraplegia
Tuberculous arachnoiditis
Non-osseous spinal tuberculoma
Spinal meningitis

The clinical manifestations are the results of complex interaction between the host defensive mechanism and virulence of the tuberculous bacilli. Basal meningeal adhesions secondary to inflammation for instance can lead to cranial nerve palsies particularly the optic, the oculomotor, the abducens and the auditory nerves [8] in 20–30% of TBM cases. Commonly the abducens nerve is involved due to its small size, long course and can act as a false localizing sign indicating increased ICP in 22% of cases [9]. Cranial neuropathy is considered the single most important neurological predictor that differentiates between acute bacterial meningitis (ABM) and TBM [10]. Cerebral infarctions likewise occur due to infiltrative exudates of the cortical and meningeal blood vessels commonly causing vasculitis, thrombosis, vasospasm, and strangulation of blood vessels affecting the internal capsule and basal ganglia leading to hemiparesis and movement disorders, respectively [11]. Ischemic stroke secondary to CNS infection such as bacterial and fungal are reported in 15–25% of cases [12] in comparison to 10–47% in TBM and it predicts poor outcome at 3 months, signifying an important morbidity factor [13]. The treatment of stroke in the context of TBM is controversial, some authors adhere to the traditional ischemic stroke treatment with anti-platelets therapy however others rely on treatment of the underlying infection alone.

In a similar process, exudates interfering with CSF flow at the basal cisterns can result in hydrocephalus which can be of the communicating or the obstructive type at a later stage [14] with the former being more common [15]. Comparably, the presence of exudates in HIV negative individual is particularly robust and attest to “over inflammation “with resultant hydrocephalus which preferentially occur in children in 70% of cases [16], acting as a poor prognostic indicator, as opposed to only 12% in adults [17]. It is seldom seen in HIV positive cases as the exudates tend to be less thick and serous as opposed to the much thicker and gelatinous exudates seen in HIV negative patients [18] such as ours. Interestingly, the presence of granuloma in TBM tells a fascinating survival battle story of between the pathogen (switching to a dormant phase) and the immune system (trying to wall off the spread of the infection). Seizure on the other hand can occur due to the former mentioned complications or as part of intra-parenchymal neuro-tuberculosis which preferentially presents with granulomas and rarely as an abscess [19] which is not the case here.

Cerebral venous thrombosis (CVST) is considered a rare complication [20]. One that is attributed to vascular endothelial damage, stasis, and hypercoagulable state secondary to infection rather than inherited thrombophilia per se which is responsible for 20% of CVST cases worldwide [21].

Our patient is special in illustrating the sum of those complications occurring concurrently. The presence of multiple complications correlates with severity of infection despite negative microbiological work up, however, the utilization of validated TBM scoring systems has allowed for early treatment improving his odds of complete recovery 5 times more compared to patients receiving late treatment [22]. Validated clinical scores such as the TDS and LCS give clinicians a supporting hand in diagnosing TBM particularly in the absence of positive cultures and have excellent accuracies [23].

## Conclusion

This report highlights the importance of complications’ pattern recognition in TBM despite the absence of microbiological confirmation. It also demonstrates the challenge in reaching the diagnosis given the low sensitivity of gold standard diagnostic tool (TB culture) despite repeated large volume CSF analysis, and the low sensitivity of CSF TB PCR. It also provides insight to the utilization of validated clinical scoring systems with Thwaites diagnostic score (TDS) being a simple screening tool and possibly utilization of the more complicated, imaging-reliant; Lancet Consensus score (LCS) as confirmatory tool for possible and probable TBM.

## Ethical approval

This case was part of an approved study by the Hamad Medical Corporation Medical Research Center, research protocol #13006/13.

## Conflict of interest

The authors report no conflict of interset.
